# Use of ustekinumab in the treatment of severe psoriasis in a liver transplant recipient^☆^

**DOI:** 10.1016/j.abd.2025.501156

**Published:** 2025-07-03

**Authors:** Tiago Almeida Santos Costa, Juliana Schinzari Palo, Renata Ferreira Magalhães

**Affiliations:** Department of Clinical Medicine, Dermatology Outpatient Clinic, Hospital Universitário da Universidade de Campinas, Campinas, SP, Brazil

Dear Editor,

Biologics represent an important advancement in the treatment of psoriasis, especially in moderate to severe cases that do not respond to conventional therapies. These agents act precisely on specific molecular targets, providing effective disease control and substantially improving patients' quality of life.^1^

In solid organ transplant recipients (OTR), the use of biologics gains even greater clinical importance. These patients, who require continuous immunosuppression to prevent graft rejection, often experience psoriasis exacerbation induced by medications like corticosteroids. Additionally, the toxicity of drugs like cyclosporine on transplanted organs and the increased risk of infections and malignancies make the management of these cases even more complex.^2^

Recent literature highlights the efficacy and safety of biologics in treating psoriasis in OTRs but emphasizes the need for a cautious approach when selecting these therapies. Documented clinical cases demonstrate that, while biologics offer significant improvement, careful patient monitoring is essential due to potential interactions with immunosuppressive drugs and the risk of serious complications.^3^, ^4^

In 2023, a study reviewed the literature on patients with psoriasis who had undergone solid organ transplantation and received biologic therapy. The analysis identified 13 cases, including 8 liver transplants, 4 kidney transplants, and 1 pancreas-kidney transplant, in which patients, primarily treated with etanercept and ustekinumab, showed positive responses without complications over periods ranging from 5 months to 6 years.^4^

The study also highlighted three specific cases: the first, a man who underwent a kidney transplant and showed significant improvement after a severe psoriasis relapse, treated with ustekinumab; the second, a liver transplant patient with severe psoriasis who responded positively to treatment with etanercept; and finally, a female kidney transplant recipient with psoriasis since childhood who achieved an excellent response after 30 weeks of treatment with risankizumab.^4^

We report the case of a 35-year-old man with a history of hypertension and mild plaque psoriasis, treated with methotrexate (15 mg weekly) for seven months, who developed acute liver failure due to cryptogenic cirrhosis, necessitating a liver transplant. Initially, after the transplant, the patient was maintained on immunosuppressive therapy with tacrolimus and mycophenolate mofetil, showing a good response in his skin condition (PASI score < 5). However, one year later, there was a progression of the disease to extensive plaques across the body and scalp, negatively impacting his quality of life.

Treatment with acitretin (50 mg/day or 0.5 mg/kg/day) provided partial improvement for three years, but there was secondary failure. Narrowband UVB phototherapy was initiated, resulting in clinical worsening with transformation to pustular psoriasis (Fig. 1), which gradually resolved with the reintroduction of acitretin. After the transplant, the patient developed obesity, chronic kidney disease, and hepatic steatosis. The elevation of serum lipids with the use of acitretin and the contraindication to methotrexate and cyclosporine due to additional complications limited therapeutic options.

**Figure 1:**
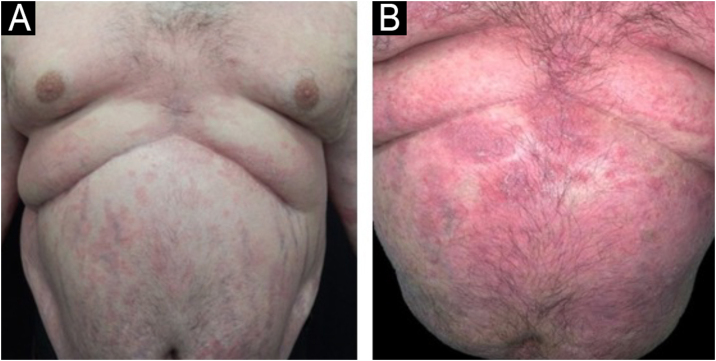
(A) Plaque psoriasis and striae on the trunk before acitretin. (B) Pustular eruption after phototherapy.

After a multidisciplinary evaluation, the patient was treated with ustekinumab, a monoclonal antibody targeting the p40 subunit common to interleukins 12 and 23. With an induction dose of 90 mg (weight 100 kg), he achieved PASI 100 after the first maintenance dose (12 weeks later). Control of psoriasis was maintained with dosing every 12 weeks for three years, although there was a recurrence of plaques on the limbs and abdomen, reaching PASI < 75. PASI 90 was again achieved by reducing the interval to 8 weeks, as dose optimization or interval reduction is recommended in the product information, without compromising treatment safety. The patient has remained well-controlled for five years, with no evidence of lesions and stable liver function (Fig. 2).

**Figure 2:**
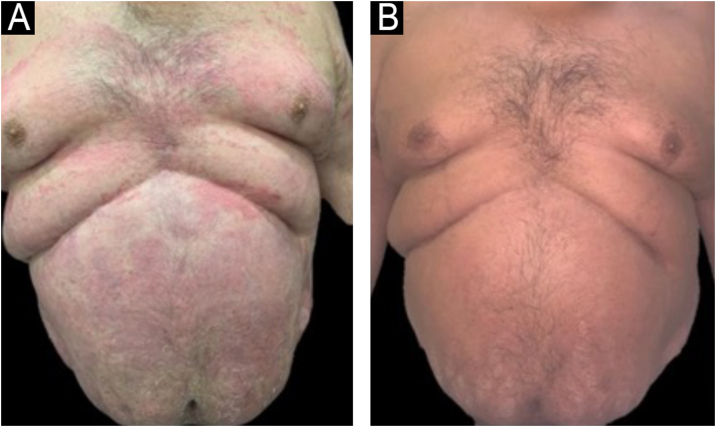
(A) Infiltrative erythematous-scaly plaques on the trunk before Ustekinumab. (B) Ten months after starting ustekinumab, no lesions present.

The risk of hepatotoxicity and the complex interactions between immunosuppressants and therapies for psoriasis are among the conditions that make treatment in organ transplant recipients challenging. Ustekinumab (UST) offers an alternative with proven safety, as it is the longest-standing anti-interleukin available in the Brazilian market and has several reports in this patient profile. However, the absence of specific guidelines and the need for individualized approaches underscore the complexity of managing this condition, highlighting the importance of further research to enhance treatment.^5^

This case reinforces that ustekinumab can be an effective and safe option for controlling psoriasis in liver transplant recipients without compromising liver function over a long period, highlighting the importance of personalized therapeutic strategies and the need for rigorous monitoring and further research.

## Financial support

None declared.

## Authors' contributions

Tiago Almeida Santos Costa: Study conception and design; data collection; manuscript drafting; critical literature review.

Juliana Schinzari Palo: Manuscript drafting; critical literature review.

Renata Ferreira Magalhães: Critical revision of important intellectual content; effective participation in research supervision; final approval of the manuscript version.

## Conflicts of interest

None declared.
